# P01-11 Economic impact of health-enhancing-physical activity from different settings in France

**DOI:** 10.1093/eurpub/ckac095.011

**Published:** 2022-08-29

**Authors:** Antoine Noël Racine, Anne Vuillemin, Bénédicte Meurisse, Jean-François Toussaint, Gautier Christèle

**Affiliations:** 1 Pôle Ressources National Sport Santé Bien-Etre, Ministry of Sports and the Olympic and Paralympic Games, Vichy, France; 2 Université Côte d'Azur, LAMHESS, France; 3 General Commission for Sustainable Development, French Ministry for the ecological transistion, Paris, France; 4 URP 7329, Université de Paris, Paris, France; 5 IRMES, INSEP, Hôtel-Dieu, APHP, Paris, France; 6 Sport Policy Development Office, French Ministry of Sports and the Olympic and Paralympic Games, Paris, France

**Keywords:** economic evaluation, physical activity, health promotion, chronic diseases, scoping review

## Abstract

**Background:**

There is strong evidence of the multiple benefits of physical activity on health in primary, secondary and tertiary prevention (WHO, 2020). However, economic evaluations are still needed to estimate direct and indirect costs that could be saved from various Health-Enhancing Physical Activity (HEPA) promotion strategies (Ding et al., 2020). Moreover, these savings might be influenced by specificities of the national health system. The aim of this study is to explore the economic impact of HEPA from different settings in France.

**Methods:**

A systematic scoping review of grey and scientific literature was conducted. Relevant articles were identified through searching from PubMed, ScienceDirect, SportDiscus databases and from google. Searches were conducted in English and French between January 2000 and December 2020. A data extraction template was used to collect, organize and summarize data regarding the following variables: aim of the study, study population, study settings, methods, and main results.

**Results:**

A total of 17 studies were included from the grey literature (n = 10) and the scientific peer-reviewed literature (n = 7). Data from each variables of interest were heterogeneous, making comparisons difficult. Studies were categorized in 5 types: studies aiming to estimate the cost of physical inactivity or the cost that could be saved from HEPA promotion in general population (n = 8); studies aiming to evaluate the medico-economic impact of a physical activity adapted program (n = 5); studies aiming to assess the economic benefit of active travel in a city (n = 3); study aiming to estimate the economic impact of physical activity from a company and its employee's perspectives (n = 1). Whatever the methods, the study population or the study setting, several tens of millions of euros to several billions of euros could be saved each year by investing in HEPA promotion.

**Conclusions:**

HEPA promotion can lead to substantial saving. Methods should be standardized to more precisely estimate its extent in different settings in France. This could help policy-makers in their decision to invest in HEPA promotion, especially in phases of epidemics, where sedentarity and physical inactivity account for major health risks.

